# Tell me all about it: Narrated memories are less emotional than imagined memories

**DOI:** 10.1177/17470218221126720

**Published:** 2022-10-07

**Authors:** Jackie Andrade, Tom IJdema, Nicola Vadasz, Jon May

**Affiliations:** 1 School of Psychology, University of Plymouth, Plymouth, Devon, UK; 2 Department of Medical and Clinical Psychology, Centre of Research on Psychology in Somatic Diseases, Tilburg University, Tilburg, Noord-Brabant, The Netherlands

**Keywords:** Autobiographical memory, mental imagery, talking therapies, emotion

## Abstract

People often re-live memories by talking about them. Verbal thinking is usually less emotive than imagery-based thinking but it is not known if this finding generalises to recollection. We tested if narrating memories aloud reduces their affective charge compared with recollecting them using imagery. Participants were randomised to two conditions: imagery (recalling the memory silently as vividly as possible) or narration (describing the memory out loud as clearly as possible). After practicing with a neutral topic, they recalled three aversive (Experiments 1 and 2) or three happy (Experiment 3) memories using narration or imagery, and rated emotionality of the memory after each recall. Before and after the procedure, they completed the PANAS to measure effects on mood. Experiments 2 and 3 included a 24 h follow-up. Emotionality was consistently lower following narrated recollection than imaginal recollection: narrated *M* = 5.3, *SD* = 2.5; imaginal *M* = 7.2, *SD* = 2.0; effect size (difference in mean values divided by overall *SD*) = 0.78. Negative affect increased after recollection of aversive memories and positive affect decreased, but there were no effects of condition upon mood. Recalling a positive memory had no effect on mood. Follow-up data showed no lasting effects of recall mode on availability of memories or mood. We conclude that narration of emotional autobiographical memories reduces the emotionality of the recollection, but does not differentially change mood compared with image-based recall.

“The recollection of what I then said, of my conduct, my manners, my expressions during the whole of it, is now, and has been many months, inexpressibly painful to me.”

Fitzwilliam Darcy, Pride and Prejudice

“Think only of the past as its remembrance gives you pleasure.”

Elizabeth Bennet, Pride and Prejudice

Recollection can be a painful or pleasurable experience, as these quotes illustrate. This article asks whether the extent to which it is so depends on the cognitive processes that are deployed during recollection.

Recollection typically involves mental imagery (Brewer & Pani, 1996), which gives a sense of immediacy and reliving (Greenberg & Knowlton, 2014). When people are instructed to recall emotional events while performing concurrent tasks that block this imagery, they typically rate their memories as less vivid and less emotional (e.g., Houben et al., 2020; Mertens et al., 2021). The assumption that recollection is image-based is also implicit in the literature on episodic future thought, which compares the cognitive and neural similarities of recollecting the past with imagining the future (e.g., Hassabis & Maguire, 2007; Schachter et al., 2012; Szpunar & Schacter, 2013). These studies suggest that autobiographical or episodic recollection is consistently image-based.

However, outside the laboratory, individuals often focus on verbal recall rather than imagery, as when someone narrates an experience during conversation or therapy. There are indications that narration aloud will reduce the emotionality of recall, from studies that directly compare verbal processing with imagery. For example, Vrana et al. (1986) found that imagining fear-provoking scenarios increased heart rate more than silently reading the same sentences. Holmes and Mathews (2005) compared the impact of imagining benign or anxiety-provoking scenarios with verbally thinking about the same material. Participants’ state anxiety scores increased more after imagery than after verbal thought about the negative scenarios. In this study, anxiety did not decline differentially after imagery of benign scenarios, a finding that may have been due to insensitivity of the state-trait anxiety inventory (STAI; Spielberger et al., 1983) to changes in positive mood. Subsequent studies used a measure specifically for positive affect—the positive items on the PANAS (Watson et al., 1988)—and found greater increases in positivity following imagery of positive but ambiguous scenarios than following verbal thinking (Holmes et al., 2006; Nelis et al., 2012). In sum, imagery of experimental scenarios is associated with greater affective change that verbal processing of the same scenarios.

If these findings generalise to autobiographical recall, we would predict a greater affective response when recollecting a memory using imagery than when talking about the same memory. There is some evidence consistent with this prediction. Nelis et al. (2015) asked participants to imagine or think verbally about a positive past event. Imaginal recollection consistently increased positive affect, whereas the results for verbal recall were inconsistent across two experiments. It is unclear from these findings if verbal recall of autobiographical memories makes them less emotive than image-based recall. However, the form of verbal thinking employed in this study differed from that used when narrating a memory. Participants were asked to talk about the meaning of the event and why it happened, or about how their life has worked out since the remembered event. The imagery condition therefore differed from the verbal condition in terms of its focus on concrete detail as well as the use of imagery per se.

Slofstra et al. (2017) addressed this confound by experimentally comparing the effects of concrete and abstract verbal thinking with imagery of autobiographical memories. In the concrete verbal thinking condition, they instructed participants to “describe in your mind” what happened, in what order, and what each person involved did or said. In the abstract verbal thinking condition, they instructed participants to think about why the event occurred, what it means, and how it has influenced them. In the imagery condition, they asked participants to “recall the memory with your mind’s eye” to see what is happening in detail and to recall other senses too. These manipulations increased the extent to which participants recalled their memories in concrete, abstract or imagery modes as instructed, but did not result in solely the desired processing mode. Slofstra et al. (2017) found no clear effect of processing instruction on change in positive and negative affect after memory recall, suggesting that findings on thinking mode with experimental stimuli do not generalise to autobiographical recall.

These findings leave something of a puzzle. It is not immediately clear why emotional autobiographical memories should be resistant to the effects of processing mode. Autobiographical memories are sensitive to concurrent working memory loads that block imagery (Lilley et al., 2009; van den Hout et al., 2001) so one would expect them also to be sensitive to processing mode manipulations that inhibit or encourage imagery. Slofstra et al.’s (2017) findings suggest they are not. We therefore wanted to consolidate the findings on autobiographical recall. This was the aim of this study. We made three important methodological changes. First, we asked participants to rate the emotionality of their memory immediately after recall, to increase the sensitivity of the study to fleeting changes in emotion that were not sufficient to change the person’s overall affective state. This change allows comparison with findings on the effects of working memory loads on emotionality. Second, we asked participants to narrate their memory aloud, rather than to think silently about it. This change moves away from the verbal thinking protocols used in Holmes and Mathews (2005) and subsequent studies, but better approximates how people naturally recall memories in conversations or talking therapies and provides greater control over the processing mode they employ. Third, we gave participants some practice at the required mode of recall, by adapting the lemon task used by Holmes and Mathews (2005). Participants either imagined or verbally narrated aloud the task of cutting up a lemon before using the same modes to recall their memories.

## Experiment 1

Experiment 1 compared verbal narration aloud with imagery-based recollection of negative autobiographical memories. On each trial, participants rated the emotionality of their memories immediately after a verbal or image-based recall period. They rated their mood at the start of the study period and again when all the recall trials were completed. Based on findings with experimenter-provided emotional scenarios, we predicted that emotionality would be higher and mood would change more in the imagery condition than in the verbal narration condition.

## Experiment 1 methods

### Participants and design

We recruited 36 psychology undergraduate students at the University of Plymouth to participate in the experiment for course credit. Ethical consent for the study was obtained from the Faculty of Science and Technology Ethics Committee at Plymouth University. The sample of participants consisted of 28 females and eight males, with a mean age of 23.06 years (*SD* = 8.03, range 18–47).

**Figure 1.**
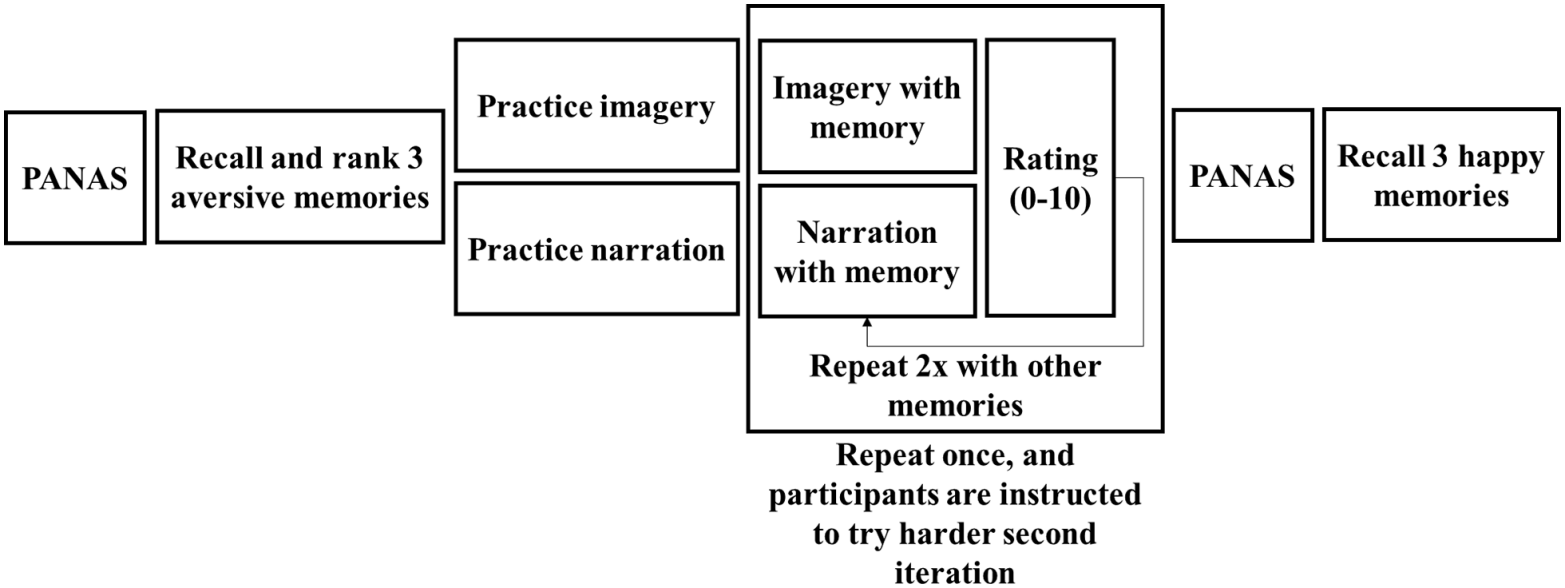
Graphical representation of the experiment.

Participants were randomly allocated to recall memories using imagery or verbal narration. In each condition, they recalled three autobiographical memories, rating emotionality and recall quality after each recall trial. They then repeated the three trials, to maximise practice at recalling with verbal or image-based processing. They rated mood before and after the whole procedure (Figure 1).

Emotionality and Recall thus have fixed within participant factors of Time (Block 1 and Block 2), while Mood has a fixed within participant factor of Time (before, after). Participants and Memory are treated as random factors. We did not have a specific prediction about the effect of time: conceivably memories would become more vivid and emotional as recall developed, or they might become less emotive through habituation, as has sometimes been observed in studies with multiple recall trials (e.g., Lilley et al., 2009 but not Kavanagh et al., 2001).

### Materials

#### Emotionality

To measure emotionality of the aversive memory, participants were asked to “rate how emotional you feel” on a scale ranging from 0 (*neutral*) to 10 (*as bad as if it was happening right now*) after each memory recall.

#### Recall quality

To increase the plausibility of the task for participants, we asked them to rate the quality of their recall. In the verbal narration condition, they rated completeness of their narration on a 10-point scale ranging from 0 (*not complete, needed more time to complete*) to 10 (*more than enough time*). In the imagery condition, they rated vividness of the recollection on a 10-point scale ranging from 0 (*no image at all*) to 10 (*a perfectly clear representation of the image*).

#### Mood

We assessed mood with the Positive and Negative Affect Schedule (PANAS; Watson et al., 1988) before and after the recall of all three negative autobiographical memories. The PANAS consists of 10 negative (e.g., distressed, upset, and hostile) and 10 positive (e.g., alert, inspired, and active) mood adjectives, rated on a 5-point scale from 1 (*not at all*) to 5 (*extremely*) indicating to what extent they feel this way right now. The two subscales have good internal consistency (Cronbach’s alpha α = .90 for PA and α = .87 for NA; Watson et al., 1988).

#### Vividness of imagery

We included a measure of participants’ vividness of imagery, the Plymouth Sensory Imagery Questionnaire (PSIQ, Andrade et al., 2014). PSIQ measures vividness of mental imagery in seven different modalities (vision, sound, smell, taste, touch, bodily sensations, and emotional feelings) with 35 items rated on a scale from 0 (*No image at present at all, you only “know” that you are thinking of the object*) to 7 (*Perfectly clear and as vivid as the actual experience*). An example visual item is “Imagine a sunset.” The PSIQ displayed excellent internal consistency in previous research: α = .96 (Andrade et al., 2014).

#### Demand characteristics check

We assessed demand effects in both conditions at the end of the study. Participants in the verbal condition were asked “How much, if at all would you predict that focusing on describing your distressing memories in words, rather than recalling them normally, would affect any negative feelings?” Participants in the imagery condition were asked “How much, if at all would you predict that focusing on imagining your distressing memories in your head, rather than recalling them normally would affect any negative feelings?.” Each was rated on a 10-point scale, where 0 indicated “very much decrease” negative feelings, 5 indicated “do nothing” and 10 indicated “very much increase” negative feelings.

### Procedure

Participants began by completing the PANAS questionnaire. Next, they recalled times when they felt scared, distressed or unhappy. Some examples were given (e.g., finding out someone close to you had died, failing an exam, and being let down by a friend). Participants then ordered their memories from least emotive to most emotive, and the three most emotive were used in the study.

Participants in the narration condition completed a practice task of verbalising the act of cutting up a lemon, focusing on including all the details of the task in the correct sequence. Participants in the imagery condition imagined cutting up a lemon, focusing on bringing to mind all the sensory details—the tangy smell, the rough skin and so on—as vividly as possible.

Next, participants put their assigned training into practice by recalling the least emotive of their three chosen negative memories for 1 min by either narrating or visualising the memory. In the narration condition, participants narrated their memory aloud into a voice recorder placed on the desk in front of them, focusing on giving a complete and accurate account of what happened. Participants in the imagery condition brought their memory to mind, reliving the experience as vividly as possible by focusing on feelings and sensations. After 1 min, participants rated the current emotional intensity of the memory and the quality of recall. This procedure was repeated for the remaining two memories, finishing with the most emotional memory.

When participants had recalled all three memories, they had a 1-min break and then repeated the task of visualising or narrating each memory, with instructions to try harder at focusing on the wording of their narrative in the verbal condition or to try harder at focusing on feelings and sensations to make the image as vivid as possible. Upon completion of the final negative memory recall, participants completed the PANAS for a second time.

Then, to counteract any negative effects on mood, participants generated three positive memories and either narrated or visualised their memories (depending on their condition). The session concluded with participants completing the PSIQ and answering the demand question.

### Data analysis

We analysed effects of recall mode by conducting a repeated measures Bayes factor analysis of variance (ANOVA) with time, condition and Time*Condition as effects and participant as a random factor. For the emotionality and recall analyses, we also included memory as a random factor. To perform the analyses, we used the anovaBF function of the BayesFactor package in R version 0.9.12-4.3 (Morey & Rouder, 2018; R Core Team, 2021). This function generates a Bayes factor (BF_10_) per model, which specifies the relative likelihood of the model being true (H_1_) compared with the base model with only random factors (H_0_). Once a model is identified, models which add or subtract effects can be evaluated by comparing the relative size of their BF_10_ (i.e., the ratio of new model: current model). A ratio above 1 is evidence for the new model, while a value below 1 can be interpreted as evidence against the new model. For example, a BF_10_ = 3 signifies that the new model is 3 times more likely than the current model, and BF_10_ = 0.33 the opposite. Although the Bayes factor is a continuous value (rather than relying on a cut-off as in tradition null hypothesis testing), researchers have formulated guidelines to assist in interpretation (Wetzels & Wagenmakers, 2012). Values between 0 and 1/3 give evidence against a model, between 1/3 and 3 are inconclusive, and above 3 support a model. Where we report effect sizes *d*, these are computed as the ratio between the difference in the mean values and the standard deviation.

## Experiment 1 results

*t*-tests comparing the two conditions showed inconclusive evidence for differences in participants’ expectations for the procedures to affect negative feelings, with both mean values around the midpoint of the scale *BF* = 0.53, Imagery *M* = 6.7 (*SD* = 2.4), Narration *M* = 5.8 (*SD* = 2.6) *d* = 0.38; and inconclusive evidence for differences between the conditions in participants’ ability to create vivid images of neutral stimuli (PSIQ) *BF* *=* 0.77, Imagery *M* *=* 3.9 (*SD* = 0.7), Narration *M* *=* 4.3 (*SD* = 0.6), *d* = 0.49. A repeated-measures ANOVA on recall quality found evidence largely in favour of the null hypothesis (0 < All *BF* *<* 0.6), indicating equivalent satisfaction with recall between conditions and over time with *M* = 6.3 (*SD* = 2.6); contrary to expectations, recall quality was not rated more highly when memories were recalled for a second time (*BF* = 0.5).

For emotionality, there was evidence in favour of including just the effect of condition (*BF* = 4.8), with evidence against adding the effect of time (*BF* = 3.0 against) or time and the interaction (*BF* = 14 against). Participants in the imagery condition displayed higher emotionality ratings *M* = 7.0, (*SD* = 2.2) than participants in the narration condition *M* = 5.3 (*SD* = 2.9), *d* = 0.62.

**Figure 2.**
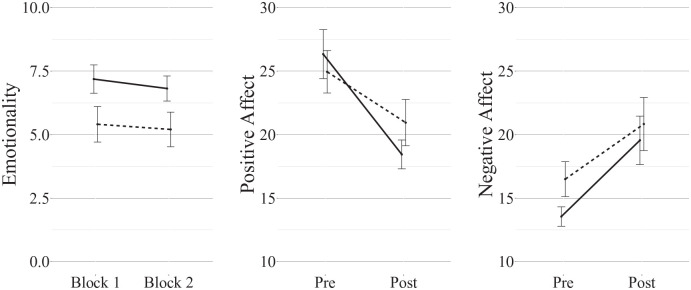
Mean (±1SE) ratings of mean memory emotionality after each block of the procedure, and of positive and negative affect before and after the whole recall procedure (solid line = imagery, dashed line = narration).

For negative affect, there was strong evidence in favour of the effect of time (*BF* = 3.6 × 10^2^), inconclusive evidence against adding an effect of condition (*BF* = 1.6 against adding) and evidence against adding both the condition and the interaction (*BF* = 4.5 against). For positive affect, there was evidence in favour of the effect of time (*BF* = 2.4 × 10^4^), and inconclusive evidence against adding an effect of condition (*BF* = 2.2 against adding) or condition and the interaction (*BF* = 1.3 against adding). Taken together, the data were best described by the effect of time as sole factor, regardless of condition. Inspection of the mean values shows that positive affect decreased and negative affect increased over time in both conditions (Figure 2).

## Experiment 1 discussion

As predicted, negative autobiographical memories were rated as more emotional after image-based recollection than verbal narration. This finding is consistent with research on the impact of concurrent tasks on emotionality (e.g., Lilley et al., 2009; van den Hout et al., 2001), where working memory tasks that impede imagery also reduce the emotionality of recollections relative to verbal loads or no-task conditions. Positive affect decreased and negative affect increased after recalling negative memories but, in contrast to predictions from research on imagined scenarios (Holmes & Mathews, 2005; Holmes et al., 2008), the changes did not seem to be dependent on processing mode. These contrasting results warranted replication.

## Experiment 2

Experiment 2 replicated the methods of experiment 1 and added a follow-up period to test whether recall mode differentially affected the availability of memories or mood after a delay. Conceivably, mood effects might take a while to emerge. There is evidence that image-based emotional memories are more accessible, more likely to intrude, than verbal memories. For example, Holmes et al. (2004) found that concurrent tasks designed to block verbal processing of a trauma film led to more reports of intrusive memories than concurrent visuospatial tasks. In a study by Halligan et al. (2002), participants who thought conceptually about what was happening in a trauma film subsequently reported fewer post-traumatic stress disorder (PTSD)-related symptoms including intrusive memories than participants who focused more on the sensory aspects of the film. These findings are consistent with a hypothesis that image-based recollection will increase availability of memories compared with verbal narration and that this increased availability will increase negative affect.

The addition of a follow-up session allowed another improvement to the design. The demand characteristic question in Experiment 1 only asked participants about the condition that they experienced. It might potentially influence ratings at follow-up, therefore In Experiment 2, we assessed demand characteristics at follow-up and did so with two questions. Participants rated their expectations of each experimental condition and did so after a neutral recall task rather than recall with narration or imagery.

## Methods experiment 2

### Participants

We recruited 32 psychology undergraduate students at the University of Plymouth to participate in the experiment for course credit. Ethical consent for the experiment was obtained from the Faculty of Science and Technology Ethics Committee at Plymouth University. The sample of participants consisted of 25 females and seven males, with a mean age of 24.8 years (*SD* *=* 8.8, range 18–47).

### Design and procedure

The initial recall trials and experimental manipulations were the same as in Experiment 1. The follow-up took place a day later. At follow-up, participants were first asked to rate, for each memory, “On a scale of 0–10, with 0 being never and 10 being constantly, how often did your memory enter your head in the past 24 hours?” to test if there were lasting effects of recall mode on availability of memories. Next, for each memory, they were asked to “Recall the memory as you would in everyday life” for a timed period of 10 s and then rate emotionality; there were no imagery or narration instructions. Then, they completed the PANAS, PSIQ, and two demand characteristics questions that were prefaced with, “We recall our memories in different ways. Sometimes we recall them in our heads to ourselves and sometimes we recall them through talking to our friends. Please rate on the following scales”: Question 1 then asked “How much, if at all, would you predict that verbally narrating your memory would affect any negative feelings?” and Question 2 asked “How much, if at all, would you predict that imagining your memory would affect any negative feelings?.” As before, participants responded on a scale of 0 (*very much decrease*) through 5 (*do nothing*) to 10 (*very much increase*).

## Experiment 2 results

A *t*-test showed no evidence for differences between the conditions in imagery ability, PSIQ: Imagery *M* = 6.8 (*SD* = 1.2), Narration *M* = 6.8 (*SD* = 0.9), *d* = .08, *BF* = 0.34. The demand characteristics questions gave mean ratings somewhat above the midpoint of 5, indicating a general expectation that the recall manipulations would increase negative feelings. A repeated measures ANOVA on the demand questions with factors of condition at Time 1 and mode of recall showed evidence for the null hypotheses (0.04 < *BF* < 0.35), with expectations that verbal narration would affect negative feelings (*M* = 6.7, *SD* = 2.5) similar to those for image-based recall (*M* = 7.1, *SD* = 1.8) *d* = .19. A repeated-measures ANOVA on recall quality at time 1, with main effects of time (Blocks 1 and 2) and condition and random effects of participant and memory gave inconclusive evidence for all models (0.38 < All *BF* < 1.72) and *M* = 6.0 (*SD* = 2.7).

**Figure 3.**
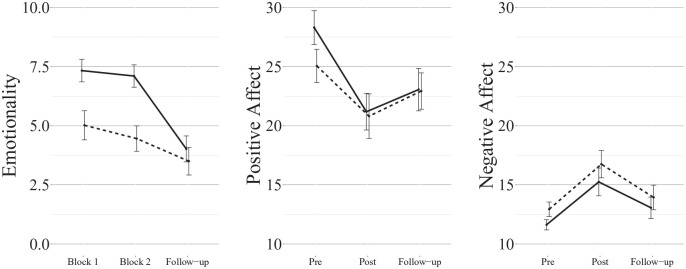
Mean (±1SE) ratings of negative autobiographical memories from Experiment 2 (solid line: imagery; dashed line: narration). Note that participants were only instructed to “think about” their memories prior to the follow-up ratings.

For emotionality of memories, we conducted a repeated-measures ANOVA with time (Blocks 1 and 2 and follow-up), condition and their interaction as fixed factors and participant and memory as random factors (Figure 3). There was strong evidence in favour of the full factorial model (*BF* = 4.3 × 10^26^), with evidence against dropping any of the terms (all *BF*s > 1.5 × 10^4^). The imagery condition again displayed higher emotionality ratings than the verbal condition, with the imagery condition decreasing to an equivalent level to the (lower) verbal score at follow-up (including only the follow-up session gave *BF* = 0.50). Repeating the analysis without the follow-up session found roughly equivalent evidence for the model with both main effects (*BF* = 162) and the model with just an effect of condition (*BF* = 144), but no interaction (*BF* = 3.7 against adding), indicating a slight decrease in emotionality in the second block and substantially higher emotionality for the imagery condition overall (*M* = 7.2, *SD* = 1.9) than the narration condition (*M* = 4.7, *SD* = 2.3), *d* = 1.01

Participants who narrated their memories reported similar availability in the following 24 hr (*M* = 3.6, *SD* = 2.5) compared with those who recollected them using imagery (*M* = 2.7, *SD* = 2.0). *d* = 0.42, BF of 0.79 shows inconclusive evidence for or against a difference but we note that numerically the mean values are in the reverse order to that predicted.

For the positive and negative affect ratings, we conducted a repeated-measures ANOVA with time and condition as main effects and participant as a random effect. For negative affect, there was evidence in favour of the effect of time (*BF* = 6.6 × 10^3^), inconclusive evidence against adding an effect of condition (*BF* = 1.64 against adding) and strong evidence against adding both the condition and the interaction (*BF* = 9.7 against). We observed a similar pattern for positive affect, with strong evidence in favour of the effect of time (*BF* = 3.2 × 10^5^), and inconclusive evidence against adding an effect of condition (*BF* = 1.9 against adding) or condition and the interaction (*BF* = 2.8 against adding). Inspection of the mean values shows that positive affect decreased and negative affect increased over time in both conditions (Figure 3). Repeating the analysis without the follow-up session gave the same pattern, replicating the lack of conclusive evidence for effects of recall mode on affect observed in Experiment 1.

## Discussion experiment 2

We replicated our findings from Experiment 1, finding increased emotionality when participants recalled autobiographical memories using mental imagery compared with when they narrated them aloud. There was no evidence that this effect was due to demand characteristics. At follow-up a day later, when participants were instructed to “think about” each memory, emotionality was similar in each condition, suggesting no lasting impact of image-based rather than verbal recollection. As in Experiment 1, there was inconclusive evidence regarding any effect of recollection mode on negative and positive affect, either immediately after recall or a day later. There was also inconclusive evidence of a difference in availability of memories after image-based or verbal recall, in contrast to previous research suggesting that imagery increases the intrusiveness of distressing material (Halligan et al., 2002; Holmes et al., 2004). However, those studies focused on the development of new trauma memories, rather than manipulating recall of existing memories. Recall mode appears to have transient effects on how much emotion participants experience while recalling a negative memory but does not affect mood or availability of the memory. Experiment 3 tested whether these results extended to positive memories.

## Experiment 3

Imagining positive experimental scenarios leads to greater increases in positive mood than verbal thinking (Holmes et al., 2006, 2008; Nelis et al., 2012; Zbozinek et al., 2015). There is less evidence on effects of imagery on positive autobiographical recall. Nelis et al. (2015) asked participants to recall positive memories and replicated the enhancing effect of imagery for positive affect, but they only used one item measures of positive and negative affect and did not report changes in negative affect. It seems that findings from the standardised paradigm are only partly replicated in positive autobiographical memory. On top of that, we know of no studies that included a follow-up measure, to see whether effects hold over time.

Our third experiment therefore tested the effects of image-based recollection versus verbal narration on positive autobiographical memories. As in Experiment 2, we assessed emotionality immediately after recall to maximise sensitivity. We used PANAS to measure positive and negative affect before and after the procedure and a day later. We also assessed memory availability over the 24-h period following the recall procedure, to test if imagery increased availability compared with verbal recall.

## Experiment 3 methods

### Participants

We recruited 41 psychology undergraduate students at the University of Plymouth to participate in the experiment for course credit. One participant failed to turn up for the follow-up therefore data analyses are based on *N* = 40. Ethical consent for the study was obtained from the Faculty of Science and Technology Ethics Committee at Plymouth University. The sample of participants consisted of 34 females and six males, with a mean age of 20.8 years (*SD* = 4.4, range 18–45).

### Design

The same design as was used in Experiment 2, except that now positive memories were used. Emotionality was rated on a scale of 0 (*neutral*) to 10 (*as good as if it was happening right now*).

## Results experiment 3

A *t*-test found no evidence of differences in imagery ability between conditions *BF* = 0.32, Imagery *M* = 5.8 (*SD* = 1.6), Narration *M* = 5.9 (*SD* = 1.1) *d* = 0.10. A repeated measures ANOVA on the demand questions with the factors of condition and mode of recall showed evidence that was inconclusive or favoured the null (0.1 < *BF* < 1.3), with expectations that verbal narration would affect positive feelings (*M* = 7.0, *SD* = 1.6) similar to expectations for image-based recall (*M* = 7.1, *SD* = 2.0), *d* = 0.03. The highest BF of 1.3 was for the effect of condition, where the verbal narration group expected a slightly higher effect overall (*M* = 7.5, *SD* = 1.4) than the imagery group (*M* = 6.6, *SD* = 2.0), *d* = 0.48, but this BF is less than the value of 3 usually taken as the criterion for a reliable difference.

A repeated measures ANOVA on recall quality with condition and time (Block 1 and Block 2) as fixed factors and participant and memory as random factors gave the same null findings as in Experiments 1 and 2, with inconclusive evidence for all effects (0.3 < All *BF* < 0.9) and *M* = 6.9 (*SD* = 2.5)

**Figure 4.**
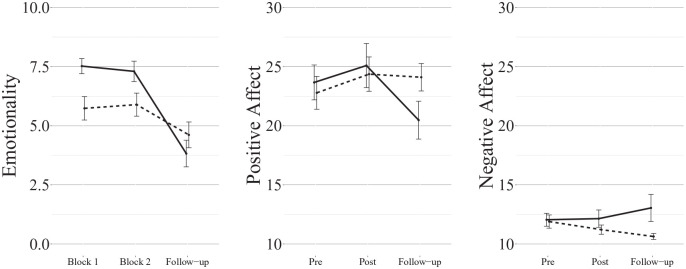
Mean (±1SE) ratings of positive autobiographical memories from Experiment 3 (solid line: imagery; dashed line: narration). Note that the memories were not imagined or narrated prior to the follow-up ratings.

For emotionality, the analysis showed strong evidence in favour of the full factorial model (*BF* = 1.2 × 10^47^), and against dropping any of the terms (all *BF*s > 1.9 × 10^11^). Inspection of the mean values shows higher emotionality in the imagery condition during the recall procedure, which dropped to the same level as the narration condition at follow-up (Figure 4). Repeating the analysis without the follow-up session showed only an effect of condition (*BF* = 21), with evidence against adding an effect of time (*BF* = 6 against) or time and an interaction (*BF* = 15 against).

Participants who narrated their memories reported similar availability in the following 24 hr (*M* = 2.3, *SD* = 2.5) compared with those who recollected them using imagery (M = 2.6, *SD* = 2.7), *d* = 0.11, with a BF of 0.23 showing evidence against an effect of condition.

For positive affect, there was inconclusive evidence for all models (0.38 < *BF* < 1.0). For negative affect there was inconclusive evidence against an effect of condition (*BF* = 0.8) but strong evidence against an effect of time (*BF* = 0.09), condition + time (*BF* = 0.07) and the full factorial model (*BF* = 0.07). Repeating both analyses without the follow-up session also found evidence against or inconclusive evidence against any effects (0.10 < *BF* < 0.69).

## Experiment 3 discussion

Using positive memories, experiment 3 replicated the finding from Experiments 1 and 2 that narrating a memory reduces its immediate emotional impact compared with image-based recollection. As in Experiment 2, this effect on emotion did not carry over into differential effects on overall mood measured immediately after recall, and there were no effects of condition on mood or availability of the memory 24 hr after the recall period. Experiments 1 and 2 found a general decrease in positive mood and increase in negative mood following recall of negative memories. This finding was not replicated with positive memories.

## Combined analysis

An advantage of a Bayesian approach is that it allows combining of evidence without type-1 error inflation (Dienes, 2011). To address concerns about small sample size, we repeated the key analyses on merged data from all three experiments, adding experiment as a fixed factor and restricting the analyses to data collected on day 1 as Experiment 1 did not include a 24-h follow-up.

Thus, for emotionality, we conducted a mixed ANOVA with time (Blocks 1 and 2), condition (narration or imagery), Experiment (1, 2 or 3) and their interactions as fixed factors and participant and memory as random factors. This analysis gave strong evidence for an effect of condition on emotionality (*BF* = 2.7 × 10^5^) and evidence against adding other effects (all BFs against adding effect > 3) apart from block, where there was inconclusive evidence (*BF* = 1.2) against adding block to the model. Across the three experiments, mean emotionality was 7.2 (*SD* 2.0) in the imagery condition and 5.3 (*SD* 2.5) in the verbal narration condition, giving a difference of 1.90 (*SD* 2.44), *d* *=* 0.78.

For negative affect, a mixed ANOVA with time (pre, post), condition (narration or imagery), experiment (1, 2, or 3) and their interactions as fixed factors and participant as random factors gave strong evidence in favour of a model with experiment and time plus their interaction (*BF* = 4.3 × 10^13^) with evidence against changing to any other model (BFs against changing all > 3 apart from inconclusive evidence against adding condition, *BF* = 2.2). Importantly for our hypothesis that imaging negative memories would lead to higher negative affect than narrating them (Experiments 1 and 2), and imaging positive memories (Experiment 3) would lead to lower, there was no evidence for switching to the full interaction model with condition (*BF* = 105 against).

The same ANOVA applied to positive affect scores gave similarly strong evidence for a model including effects of Time and an interaction of Experiment × Time (*BF* = 4.4 × 10^9^) and no evidence for switching to other models (all BFs against adding > 3), apart from a model adding condition and a Condition × Time interaction, where there was inconclusive evidence against switching (*BF* = 2.2). There was no support for our prediction that imaginal recall would increase positive affect over time in Experiment 3 (positive memories) and decrease it in Experiments 1 and 2 (negative memories) relative to verbal narration: *BF* = 16.5 against adding condition and its full interaction with time and experiment.

## General discussion

We have evaluated the emotional effects of imagining or narrating autobiographical memories. With negative and positive memories, we found that verbally narrating a memory led to lower ratings of emotionality than imaginal recollection, but did not differentially impact mood after the recall period. This finding was not confounded by quality of recall or demand characteristics: participants in the two conditions rated their recall as similarly complete or vivid, and had similar a priori expectations about how their allocated condition would affect their mood. The immediate effect of recall mode on emotion is consistent with Vrana et al.’s (1986) finding that imagining experimenter-provided scenarios increases physiological responses more than silently reading them. It is also consistent with the many Eye Movement Desensitization and Reprocessing (EMDR)-related laboratory studies showing that concurrent tasks such as side-to-side eye movements that reduce the vividness of autobiographical memories also reduce their emotionality (e.g., Barrowcliff et al., 2004; Mertens et al., 2021; Smeets et al., 2012, for meta-analytic evidence on the efficacy of EMDR, see Lewis et al., 2020 and Seidler & Wagner, 2006; for evidence supporting the contribution of eye movements to EMDR effects, see Lee & Cuijpers, 2013).

The finding that participants rated their memories as less emotional after narration than after imagery is consistent with two somewhat different interpretations. Either narration constitutes a different form of recall compared with imagery, or it adds an additional cognitive load to that of “normal” recall that is image-based. The first interpretation is consistent with evidence from work by Holmes and others that imagery is more tightly associated with emotion than verbal thinking is (e.g., Holmes & Mathews, 2005). Imagery recreates the sensory details and feelings associated with an event in a way that verbal cognition does not; the rememberer can choose either method of recall, with different consequences for how emotional the memory feels during recall.

An alternative explanation is that imagery is the normal mode of thinking and verbal narration constitutes a cognitive load that reduces any emotional impact. Consistent with this explanation, there is evidence that autobiographical recollection typically involves imagery (Aydin, 2018; Brewer & Pani, 1996; Greenberg & Knowlton, 2014; Rubin, 2020). It is conceivable that our imagery instruction approximated “normal” recall and that the narration instruction decreased emotionality by interfering with recall. Data from the follow-up phase in Experiments 2 and 3 hint that this was not the case. Participants were asked at follow-up to “Recall the memory as you would in everyday life” for 10-s before rating emotionality. Ratings were similar to (Experiment 2) or lower than (Experiment 3) those in the narration condition. In other words, normal recall looked more like narration than like imagery at follow-up, suggesting that the imagery instruction was beneficial rather than the narration instruction being detrimental. However, an important caveat is that participants had only 10 s for recall at follow-up, because we wanted to get ratings of a more naturalistic, spontaneous recollection, compared with 60 s in the experimental phase. The relatively low emotionality ratings at follow-up may have been due to lack of time to recall the memory in detail rather than lack of imagery. Future research should compare the resource demands of image-based and verbal recall in terms of their impact on neutral comparison task, and include a “normal recall” condition to test fully whether narration decreases emotionality of memories or imagery instructions increase it against this standard. In doing this, care should be taken not to introduce imagery demands. In this study, participants in the narration condition rated the quality of their recollection in terms of completeness rather than vividness to avoid introducing a demand to imagine as well as narrate. In sum, the findings show that narrating a memory reduces immediate emotion compared with imaginal recollection, but future research is needed to explain the underlying mechanism.

We found no evidence that the greater emotionality resulting from imagery instructions had any effect upon positive or negative affect, even when data were combined across all three experiments to maximise power. No effects on mood or memory availability emerged at 24-hr follow-up. These results contrast with those from studies that used Holmes and Mathews’ approach of asking participants to imagine or think verbally about experimenter-provided scenarios (i.e., Holmes & Mathews, 2005; Holmes et al., 2008; Nelis et al., 2012; Slofstra et al., 2017; Vrana et al., 1986; Werner-Seidler & Moulds, 2012). These studies consistently report an increase in affect matching the affective content of the stimuli, that is, imagining negative scenarios increased negative affect more than verbally thinking about them. One reason for this contrast could be that participants in the narration condition of our studies employed imagery, despite instructions focusing on the wording and the physical act of narrating their memory. Although having to narrate a memory is likely to suppress imagery, it is conceivable that not all imagery would be suppressed. The same argument could be made for the condition employed by Holmes and Mathews of “thinking verbally” about a scenario. However, an important difference is that, in the case of memories, participants might generate an image from the memory to guide their narration, whereas in scenario-based protocols, participants are listening to the scenarios and do not need to recall a representation in the verbal condition. It is thus conceivable that the shift in methods from listening and thinking to verbally narrating explains the different results.

Against the explanation that verbal narration was a weaker manipulation than verbal thinking is the fact that we demonstrated a strong effect of recall condition on immediate emotionality ratings. Also, recall of negative memories produced increases in negative affect and decreases in positive affect. This finding shows that the PANAS ratings were sensitive to recall of emotional material, just not differentially so for verbal narration versus imaginal recall. Recalling positive memories in Experiment 3 did not affect PANAS ratings, a point that we return to later, but the general finding that verbal narration produced lower immediate ratings of emotionality speaks against it being a weak manipulation contaminated by imagery.

An alternative explanation for the contrast between our findings and those from previous studies is that autobiographical memories behave differently, in terms of their effects on mood, from cognitions about experimental scenarios. Other findings corroborate ours in suggesting that autobiographical recall is different. Nelis et al. (2015) and Slofstra et al. (2017) asked participants to think verbally about emotional autobiographical memories or recall them using imagery. Neither found clear differential effects of processing instruction on change in affect. In contrast, Werner-Seidler and Moulds (2012) found that participants who were instructed to think in an abstract way about the causes, meaning and consequences of a positive memory reported less reduction in sad mood than those instructed to view the memory unfolding with their mind’s eye. However, a subsequent study (Werner-Seidler & Moulds, 2014) that used an experimenter-guided protocol to reinforce the different processing modes found no differential effects on mood. Participants who had recovered from depression or never had depression reported a general decrease in sad mood ratings that had been experimentally induced, but not differential effects of recall mode. Participants with current depression, and natural rather than induced sad mood, experienced no benefits of recalling a positive memory. It was therefore unclear whether the reduction in sad mood reflected a benefit of recalling a positive memory for some participants, or a fading of the mood induction. In sum, despite finding a consistent difference in emotionality ratings after verbal narration or imaginal recollection, our findings add to a general finding in the literature that it is harder to alter mood by manipulating how a memory is processed than by manipulating how a novel experimental scenario is processed.

This analysis raises the question of why image-based processing of autobiographical memories does not differentially affect mood, compared with verbal processing, in the way that image-based processing of experimental scenarios does. One explanation, suggested by Slofstra et al. (2017), is that focusing on self-selected autobiographical memory introduces more heterogeneity in the data and might have masked differential effects on mood. However, heterogeneity did not prevent us demonstrating clear differential effects on emotionality and a general worsening of mood following recall of negative memories. An alternative explanation is that, unlike novel experimenter-provided materials, autobiographical memories are strongly interwoven in what Conway and Pleydell-Pearce (2000) called the “self memory system.” While conscious recall of a memory may cause a transient change in experienced emotion, as we observed, this network of links with other memories and autobiographical knowledge may have an ongoing influence on personality and mood. If mood is the sum of these memory influences, and if memories are richly encoded in sensory as well as abstract detail, then briefly recalling a single memory in a particular way is unlikely to have a lasting effect. Even though a memory is verbally narrated during recall, associated images and connections may exert a stabilising influence on mood even if they are not currently the focus of attention. Consistent with this suggestion, Holmes et al. (2008) found evidence for a mediating effect of autobiographical memory in the standardised paradigms. This explanation is speculative, but it suggests that the critical factor determining whether image-based processing affects mood more than verbal processing is the novelty of the material being processed. When new material is processed, as in the studies using emotional scenarios or the trauma-film paradigm, it is encoded only in the instructed verbal or image-based format, and there are no versions of the memory in other formats to modify its influence. When an existing memory is brought into awareness, links to multiple representations of the memory and to related memories are activated and these may dilute the effect on mood of the specific processing mode employed during recall.

Other differences of a methodological nature between experiments need to be recognised. First is the use of filler tasks. For example, Holmes and Mathews (2005) used a verbal filler task and Holmes et al. (2006) used a music task, specifically to allow any mood changes to dissipate, before the anxiety ratings. We did not use filler tasks; therefore, we may have experienced effects on mood that were a carryover of the transient effect on emotionality. However, this confound would have worked in the direction of increasing a differential impact of recall mode on mood rather than decreasing it. Studies with experimenter-provided scenarios have generally had a verbal component in the imagery condition as well, for example, when participants listened to descriptions and tried to imagine them in Holmes and Mathews (2005). This raises the question whether the effects found in the imagery condition in these studies can be ascribed purely to imagery, or rather to a general “net” effect of image-based and verbal processing combined (akin to Paivio’s, 1971, dual coding explanation of the memory superiority of concrete vs abstract nouns). This explanation is not entirely satisfactory as Holmes et al. (2008, 2009) confirmed the superiority of imagery over verbal processing in a more sophisticated paradigm, where participants were required to combine words with pictures that bypassed verbal descriptions. These are experimental constraints bound to working with standardised material, and did not affect our study because we used autobiographical memory which the participants generated themselves. A third difference from another study (Slofstra et al., 2017) with a null result is that we trained participants in their respective recall modes using the lemon exercises and gave them time (1 min) to imagine or narrate their memory as opposed to having participants rate their memory directly after its presentation. This difference should have increased our chance of finding a differential effect of processing style on mood. A fourth, important, difference is that we asked participants to verbally narrate their memories aloud and in detail rather than to “think verbally” about them. This condition encouraged a concrete level of processing similar to that of the imagery condition, whereas some previous research compared abstract processing with concrete imagery (e.g., Nelis et al., 2015). The requirement to speak aloud was ecologically valid, approximating how people narrate their memories in conversation, and hopefully plausible: we included a voice recorder to reinforce participants’ focus on the quality of their narration.

Although we demonstrated greater emotionality with image-based recall compared with verbal narration for both positive and negative memories, only negative memories showed a lasting impact on mood measured at the end of the recall period. In general terms, it is recognised that negative material is more potent and impactful than positive material (Baumeister et al., 2001). However, we feel the most likely explanation for the present results is a scaling effect. Our sample reported relatively high baseline positive mood scores and low negative scores. This means that there was more scope for recall of negative memories to reduce positive mood and increase negative mood than for recall of positive memories to make high positive mood scores even higher and low negative scores even lower.

These findings have implications for clinical interventions, suggesting that titrating verbal and image-based recall can be a useful way to manage a person’s level of distress during treatment sessions. Some recent interventions show lasting benefits for behaviour and mood from adding imagery to an essentially verbal process, for example, to enhance the power of motivational interviewing (Solbrig et al., 2019) or cognitive behavioural therapy (Holmes et al., 2007). In these interventions, imagery manipulations create new representations or substantially modify old ones, so they are more akin to laboratory paradigms using experimental scenarios than those with autobiographical memories. What we have shown is that changing how people recall an emotional autobiographical memory has transient effects on emotion, raising the possibility of using recall mode to induce or dampen emotion while working with memories of trauma. Analogue studies of EMDR suggest that eye movement tasks that reduce the emotionality of recollections can provide a step in exposure-based treatments for trauma (Kavanagh et al., 2001). In a similar way, simply asking the client to verbalise their memory aloud may reduce the immediate distress its recollection causes while allowing therapeutic work with the memory to progress.

In conclusion, narrating emotional memories aloud transiently reduces their emotional impact but does not have a lasting impact on mood or memory availability.

## References

[bibr1-17470218221126720] Andrade J. May J. Deeprose C. Baugh S. J. Ganis G. (2014). Assessing vividness of mental imagery: The Plymouth Sensory Imagery Questionnaire. British Journal of Psychology, 105(4), 547–563. 24117327 10.1111/bjop.12050

[bibr2-17470218221126720] Aydin C. (2018). The differential contributions of visual imagery constructs on autobiographical thinking. Memory, 26(2), 189–200. 29295687 10.1080/09658211.2017.1340483

[bibr3-17470218221126720] Barrowcliff A. L. Nicola S. G. Tom C. A. Malcolm J. (2004). Eye-movements reduce the vividness, emotional valence and electrodermal arousal associated with negative autobiographical memories. Journal of Forensic Psychiatry and Psychology, 15, 325–345. 10.1080/14789940410001673042

[bibr4-17470218221126720] Baumeister R. F. Bratslavsky E. Finkenauer C. Vohs K. D. (2001). Bad is stronger than good. Review of General Psychology, 5(4), 323–370.

[bibr5-17470218221126720] Brewer W. F. Pani J. R. (1996). Reports of mental imagery in retrieval from long-term memory. Consciousness and Cognition, 5, 265–287. 8906404 10.1006/ccog.1996.0019

[bibr6-17470218221126720] Conway M. A. Pleydell-Pearce C. W. (2000). The construction of autobiographical memories in the self-memory system. Psychological Review, 107(2), 261–288. 10789197 10.1037/0033-295x.107.2.261

[bibr7-17470218221126720] Dienes Z. (2011). Bayesian versus orthodox statistics: Which side are you on?. Perspectives on Psychological Science, 6(3), 274–290. 26168518 10.1177/1745691611406920

[bibr8-17470218221126720] Greenberg D. L. Knowlton B. J. (2014). The role of visual imagery in autobiographical memory. Memory & Cognition, 42(6), 922–934. 24554279 10.3758/s13421-014-0402-5

[bibr9-17470218221126720] Halligan S. L. Clark D. M. Ehlers A. (2002). Cognitive processing, memory, and the development of PTSD symptoms: Two experimental analogue studies. Journal of Behavior Therapy and Experimental Psychiatry, 33(2), 73–89. 12472172 10.1016/s0005-7916(02)00014-9

[bibr10-17470218221126720] Hassabis D. Maguire E. A. (2007). Deconstructing episodic memory with construction. Trends in Cognitive Sciences, 7, 299–306. 10.1016/j.tics.2007.05.001 17548229

[bibr11-17470218221126720] Holmes E. A. Arntz A. Smucker M. R. (2007). Imagery rescripting in cognitive behaviour therapy: Images, treatment techniques and outcomes. Journal of Behavior Therapy and Experimental Psychiatry, 38(4), 297–305. 18035331 10.1016/j.jbtep.2007.10.007

[bibr12-17470218221126720] Holmes E. A. Brewin C. R. Hennessy R. G. (2004). Trauma films, information processing, and intrusive memory development. Journal of Experimental Psychology: General, 133, 3–22. 14979748 10.1037/0096-3445.133.1.3

[bibr13-17470218221126720] Holmes E. A. Lang T. J. Shah D. M. (2009). Developing interpretation bias modification as a “cognitive vaccine” for depressed mood: Imagining positive events makes you feel better than thinking about them verbally. Journal of Abnormal Psychology, 118(1), 76–88. 19222316 10.1037/a0012590

[bibr14-17470218221126720] Holmes E. A. Mathews A. (2005). Mental imagery and emotion: A special relationship?. Emotion, 5(4), 489–497. 16366752 10.1037/1528-3542.5.4.489

[bibr15-17470218221126720] Holmes E. A. Mathews A. Dalgleish T. Mackintosh B. (2006). Positive interpretation training: Effects of mental imagery versus verbal training on positive mood. Behaviour Therapy, 37, 237–247. 10.1016/j.beth.2006.02.002 16942975

[bibr16-17470218221126720] Holmes E. A. Mathews A. Mackintosh B. Dalgleish T. (2008). The causal effect of mental imagery on emotion assessed using picture-word cues. Emotion, 8(3), 395–409. 18540755 10.1037/1528-3542.8.3.395

[bibr17-17470218221126720] Houben S. T. Otgaar H. Roelofs J. Merckelbach H. Muris P. (2020). The effects of eye movements and alternative dual tasks on the vividness and emotionality of negative autobiographical memories: A meta-analysis of laboratory studies. Journal of Experimental Psychopathology, 11(1), Article 2043808720907744. 10.1177/2043808720907744

[bibr18-17470218221126720] Kavanagh D. J. Freese S. Andrade J. May J . (2001). Effects of visuospatial tasks on desensitization to emotive memories. British Journal of Clinical Psychology, 40(3), 267–280. 10.1348/014466501163689 11593955

[bibr19-17470218221126720] Lee C. W. Cuijpers P. (2013). A meta-analysis of the contribution of eye movements in processing emotional memories. Journal of Behavior Therapy and Experimental Psychiatry, 44(2), 231–239. 23266601 10.1016/j.jbtep.2012.11.001

[bibr20-17470218221126720] Lewis C. Roberts N. P. Andrew M. Starling E. Bisson J. I. (2020). Psychological therapies for post-traumatic stress disorder in adults: Systematic review and meta-analysis. European Journal of Psychotraumatology, 11(1), Article 1729633. 10.1080/20008198.2020.1729633 PMC7144187 32284821

[bibr21-17470218221126720] Lilley S. A. Andrade J. Turpin G. Sabin-Farrell R. Holmes E. A. (2009). Visuospatial working memory interference with recollections of trauma. British Journal of Clinical Psychology, 48(3), 309–321. 10.1348/014466508X398943 19187579

[bibr22-17470218221126720] Mertens G. Lund M. Engelhard I. M. (2021). The effectiveness of dual-task interventions for modulating emotional memories in the laboratory: A meta-analysis. Acta Psychologica, 220, Article 103424. 10.1016/j.actpsy.2021.103424 34619553

[bibr23-17470218221126720] Morey R. D. Rouder J. N. (2018). Package “BayesFactor..” https://cran.r-project.org/web/packages/BayesFactor/BayesFactor.pdf

[bibr24-17470218221126720] Nelis S. Holmes E. A. Palmieri R. Bellelli G. Raes F. (2015). Thinking back about a positive event: The impact of processing style on positive affect. Frontiers in Psychiatry, 6, Article 3. 25806003 10.3389/fpsyt.2015.00003 PMC4353183

[bibr25-17470218221126720] Nelis S. Vanbrabant K. Holmes E. A. Raes F. (2012). Greater positive affect change after mental imagery than verbal thinking in a student sample. Journal of Experimental Psychopathology, 3(2), 178–188. 26457173 10.5127/jep.021111 PMC4599135

[bibr26-17470218221126720] Paivio A. (1971). Imagery and verbal processes. Holt, Rinehart, and Winston.

[bibr27-17470218221126720] R Core Team (2021). R: A language and environment for statistical computing. R Foundation for Statistical Computing, Vienna, Austria. https://www.R-project.org/

[bibr28-17470218221126720] Rubin D. C. (2020). The ability to recall scenes is a stable individual difference: Evidence from autobiographical remembering. Cognition, 197, 104164. 31918237 10.1016/j.cognition.2019.104164

[bibr29-17470218221126720] Schachter D. Addis D. R. Hassabis D. Martin V. C. Spreng R. N. Szpunar K. K. (2012). The future of memory: Remembering, imagining, and the brain. Neuron, 76, 677–694. 10.1016/j.neuron.2012.11.001 23177955 PMC3815616

[bibr30-17470218221126720] Seidler G. H. Wagner F. E. (2006). Comparing the efficacy of EMDR and trauma-focused cognitive-behavioral therapy in the treatment of PTSD: A meta-analytic study. Psychological Medicine, 36(11), 1515–1522. 16740177 10.1017/S0033291706007963

[bibr31-17470218221126720] Slofstra C. Eisma M. C. Holmes E. A. Bockting C. L. Nauta M. H. (2017). Rethinking a negative event: The affective impact of ruminative versus imagery-based processing of aversive autobiographical memories. Frontiers in Psychiatry, 8, Article 82. 10.3389/fpsyt.2017.00082 PMC5447674 28611690

[bibr32-17470218221126720] Smeets M. A. Dijs M. W. Pervan I. Engelhard I. M. van den Hout M. A. (2012). Time-course of eye movement-related decrease in vividness and emotionality of unpleasant autobiographical memories. Memory, 20, 346–357. 10.1080/09658211.2012.665462. 22537073

[bibr33-17470218221126720] Solbrig L. Whalley B. Kavanagh D. J. May J. Parkin T. Jones R. Andrade J. (2019). Functional imagery training versus motivational interviewing for weight loss: A randomised controlled trial of brief individual interventions for overweight and obesity. International Journal of Obesity, 43, 883–894. 30185920 10.1038/s41366-018-0122-1

[bibr34-17470218221126720] Spielberger C. D. (1983). Manual for the State-Trait Anxiety Inventory (Form Y). Consulting Psychologist Press.

[bibr35-17470218221126720] Szpunar K. K. Schacter D. L. (2013). Get real: Effects of repeated simulation and emotion on the perceived plausibility of future experiences. Journal of Experimental Psychology: General, 142(2), 323–327. 10.1037/a0028877 22686637 PMC3461111

[bibr36-17470218221126720] van den Hout M. Muris P. Salemink E. Kindt M . (2001). Autobiographical memories become less vivid and emotional after eye movements. British Journal of Clinical Psychology, 40, 121–130. 10.1348/014466501163571 11446234

[bibr37-17470218221126720] Vrana S. R. Cuthbert B. N. Lang P. J. (1986). Fear imagery and text-processing. Psychophysiology, 23(3), 247–253. 10.1111/j.1469-8986.1986.tb00626.x 3749404

[bibr38-17470218221126720] Watson D. Clark L. A. Tellegen A. (1988). Development and validation of a brief measure of positive and negative affect. Journal of Personality and Social Psychology, 54, 1063–1070. 3397865 10.1037//0022-3514.54.6.1063

[bibr39-17470218221126720] Werner-Seidler A. Moulds M. L. (2012). Mood repair and processing mode in depression. Emotion, 12(3), 470–478. 22023367 10.1037/a0025984

[bibr40-17470218221126720] Werner-Seidler A. Moulds M. L. (2014). Recalling positive self-defining memories in depression: The impact of processing mode. Memory, 22(5), 525–535. 23758472 10.1080/09658211.2013.801494

[bibr41-17470218221126720] Wetzels R. Wagenmakers E. J. (2012). A default Bayesian hypothesis test for correlations and partial correlations. Psychonomic Bulletin & Review, 19(6), 1057–1064. 10.3758/s13423-012-0295-x 22798023 PMC3505519

[bibr42-17470218221126720] Zbozinek T. D. Holmes E. A. Craske M. G. (2015). The effect of positive mood induction on reducing reinstatement fear: Relevance for long term outcomes of exposure therapy. Behaviour Research and Therapy, 71, 65–75. 26073498 10.1016/j.brat.2015.05.016 PMC4508344

